# Editorial: Microbial sensing to control host immune responses

**DOI:** 10.3389/fmicb.2022.1054640

**Published:** 2022-10-11

**Authors:** Ibrahim M. Sayed, Asima Bhattacharyya, Soumita Das

**Affiliations:** ^1^Department of Biomedical and Nutritional Sciences, University of Massachusetts Lowell, Lowell, MA, United States; ^2^Department of Medical Microbiology and Immunology, Faculty of Medicine, Assiut University, Asyut, Egypt; ^3^National Institute of Science Education and Research (NISER) Bhubaneswar, An OCC of Homi Bhabha National Institute, Khordha, Odisha, India; ^4^Department of Pathology, School of Medicine, University of California, San Diego, San Diego, CA, United States

**Keywords:** biofilm, host microbe interaction, immune response, microbial sensors, pathogen, quorum sensing, signaling pathway

The host immune system is important in controlling infections mediated by pathogens. The innate immune system of mammals gradually developed microbial sensors such as receptors and mediators that are capable of interacting with the pathogen-associated molecular patterns (PAMPs) of bacteria and controlling the downstream inflammatory processes. The expression of the microbial sensors is programmed by the microbes and the environment, which critically modulate the disease pathogenesis. In this special issue, four articles have been published that cover the role of microbial sensing in defining the host immune response, subsequent signaling pathways and cytokine profiles that determine hemostasis or disease.

The host defense produces distinct responses against commensals and pathogens, though they might express the same bacterial structure, lipopolysaccharide (LPS) (Sayed et al., 2021). In their opinion article, Tiruvayipati et al. described how enteric pathogens and non-pathogens interact differentially with the gut mucosa to either establish symbiosis or infection. The gut microbiota attaches to the host through ligand-receptor binding and the subsequent steps control the fate of the interaction. During symbiosis, commensals attach to the mucus layer of the gut through the microbe-associated molecular patterns (MAMPs) and the mucus layer prevents the invasion of other pathogenic bacteria. The changes in the gut environment facilitate the switching of commensals to pathogens, and the pathogenic bacteria mediate the infection process through pathogen-associated molecular patterns (PAMPs). Importantly, the polymorphisms in the host receptors such as Toll-like receptors (TLRs), NOD-like receptors (NLRs), C-type lectin receptors (CTLRs), and the retinoic acid-inducible gene I (RIG-I)-like receptors (RLRs) are the key players that regulate the commensals to pathogen transition.

The communication between bacteria inside a community is mediated by quorum sensing (QS), which also facilitates hijacking the host immune responses by pathogens and establishing the infection. Boopathi et al. discussed interbacterial communication and microbial behaviors during the establishment of mycobacterial pathogenesis. Although heterogenic mycobacterial populations with different resistance patterns are recorded, these populations communicate with each other through quorum sensing (QS). The strategies include the production of biofilms, siderophores, and virulence proteins. Additionally, the QS signals allow the mycobacteria to communicate with neighboring cells. The QS are formed by the subclones and then transferred to the surrounding community. Since the proteins/regulators that participate in the QS signals are preserved among *Mycobacteria*, inhibitors of this axis could decrease the pathogenesis of *Mycobacteria* and can be effective anti-mycobacterial agents. Holban et al. reviewed and compared the pathogenesis of two respiratory tract pathogens: *Bordetella* spp. and *Pseudomonas aeruginosa*. The former bacteria are the ideal models for studying the host-pathogen interactions, while *Pseudomonas* is a good model for studying the QS signaling and interbacterial communications. These pathogens utilize a two-component system (TCA) to detect and respond to the host signals. Most of the TCA of *Pseudomonas aeruginosa* are well characterized, while the TCA of *Bordetella* spp. is not completely known. In Table 1 presented in Holban et al., the authors summarized the functions of TCA for these pathogens. They colonize the host via various subsequent strategies including the secretion the bacterial secretion systems. Interestingly, six secretion systems were characterized for *P. aeruginosa*. Importantly, the type 3 secretion system (T3SS) is involved in acute infection, the type 4 secretion system (T4SS) plays a role in the production of bacterial biofilm and progression to chronic infection, while the type 6 secretion system (T6SS) is crucial for QS signaling and host-pathogen communication. Interestingly, studies correlate the pathogenicity of *Pseudomonas* to the T6SS which mediates biofilm formation (Li et al., 2020). Therefore, it becomes a target for antimicrobial agents (Mishra et al., 2022). In *Bordetella* spp., T3SS is mainly expressed and involved in colonization as well as the hijacking of the host immune responses. Besides the TCA, *P. aeruginosa* and *Bordetella* spp., produce the sigma factor, small RNAs, and chaperones which regulate virulence factors and aid in bacterial pathogenesis. *P. aeruginosa* produces alarmones such as guanosine tetraphosphate and guanosine pentaphosphate which help to develop bacterial tolerance to oxidative stress, and biofilm formation. However, the role of alarmones in the pathogenesis of *Bordetella* spp. is not completely known. The authors concluded that the study of the immunoregulatory factors produced by these bacteria is crucial for vaccine development and improving the currently available antimicrobial agents.

Microbial infections can lead to inflammatory diseases and/or autoimmune diseases. Chu et al. performed a retrospective study using the Taiwan National Health Insurance Research Database to assess the correlation between microbial infection (*Mycoplasma pneumoniae*) and the risk of developing an inflammatory disease, Systemic Lupus Erythematosus (SLE). By comparing *M. pneumoniae*-infected hospitalized patients with uninfected patients, the authors found that the risk of SLE development is significantly increased with *M. pneumoniae* infection and older patients (above 65 years) have the highest risk. Moreover, there is an increased risk of SLE (around two-fold) in 2–5-year-old children after *M. pneumonae* infection. These findings are applicable to both genders and are independent of the comorbidity factors. The authors recommended that clinicians take the appropriate measures to prevent or reduce the incidence of SLE associated with *M. pneumoniae* infection, especially in the risk group.

## Conclusion

The mode of interaction between microbes/products with the host immune cell receptors/sensors and the subsequent signaling pathways determines the fate of the infection. Therefore, the screening of microbial infections as well as the study of host immune responses should be given importance in public health policy-making to provide better integrative care to patients of infectious diseases and also to reduce the risk of developing infection-induced inflammatory autoimmune diseases.

## Author contributions

IS wrote the original draft. AB and SD reviewed and accepted the draft. All authors contributed to the article and approved the submitted version.

## Funding

SD was supported by an award from the Leona M. and Harry B. Helmsley Charitable Trust, and by NIH1R01DK107585, 1R01AI155696, and R56 AG069689. Institutional funding by NISER-Bhubaneswar (Department of Atomic Energy, India) to AB.

## Conflict of interest

The authors declare that the research was conducted in the absence of any commercial or financial relationships that could be construed as a potential conflict of interest.

## Publisher's note

All claims expressed in this article are solely those of the authors and do not necessarily represent those of their affiliated organizations, or those of the publisher, the editors and the reviewers. Any product that may be evaluated in this article, or claim that may be made by its manufacturer, is not guaranteed or endorsed by the publisher.
